# Understanding advance care planning within the South Asian community

**DOI:** 10.1111/hex.12531

**Published:** 2017-03-10

**Authors:** Patricia D. Biondo, Rashika Kalia, Rooh‐Afza Khan, Nadia Asghar, Cyrene Banerjee, Debbie Boulton, Nancy Marlett, Svetlana Shklarov, Jessica E. Simon

**Affiliations:** ^1^ Advance Care Planning Collaborative Research and Innovation Opportunities Program (ACP CRIO) University of Calgary Calgary AB Canada; ^2^ Patient and Community Engagement Research Program (PaCER) University of Calgary Calgary AB Canada; ^3^ Department of Community Health Sciences University of Calgary Calgary AB Canada; ^4^ Departments of Oncology, Medicine, and Community Health Sciences University of Calgary Calgary AB Canada

**Keywords:** advance care planning, minority groups, patient engagement, patient engagement research, qualitative research

## Abstract

**Background:**

Advance care planning (ACP) is a process of reflection on and communication of a person's future health‐care preferences. Evidence suggests visible minorities engage less in ACP. The South Asian ethnic group is the largest visible minority group in Canada, and information is needed to understand how ACP is perceived and how best to approach ACP within this diverse community.

**Objective:**

To explore perspectives of South Asian community members towards ACP.

**Design:**

Peer‐to‐peer inquiry. South Asian community members who graduated from the Patient and Community Engagement Research programme (PaCER) at the University of Calgary utilized the PaCER method (SET, COLLECT and REFLECT) to conduct a focus group, family interviews and a community forum.

**Setting and participants:**

Fifty‐seven community‐dwelling men and women (22‐86 years) who self‐identified with the South Asian community in Calgary, Alberta, Canada.

**Results:**

The concept of ACP was mostly foreign to this community and was often associated with other end‐of‐life issues such as organ donation and estate planning. Cultural aspects (e.g. trust in shared family decision making and taboos related to discussing death), religious beliefs (e.g. fatalism) and immigration challenges (e.g. essential priorities) emerged as barriers to participation in ACP. However, participants were eager to learn about ACP and recommended several engagement strategies (e.g. disseminate information through religious institutions and community centres, include families in ACP discussions, encourage family physicians to initiate discussions and translate materials).

**Conclusions:**

Use of a patient engagement research model proved highly successful in understanding South Asian community members' participation in ACP.

## 1 INTRODUCTION

Advance care planning (ACP) is a process of reflection on and communication of a person's future health‐care preferences. Best viewed as a process, not an event, ACP encourages on‐going dialogue between a patient, his or her family, and the health‐care team that can guide medical decision making, even when a person becomes incapable of consenting to or refusing health care. ACP has been associated with improved adherence to patient wishes, improved patient quality of life and death, less stress and bereavement for families, and less resource‐intensive treatment at the end of life.^1^, ^2^, ^3^

At its core, ACP is based on a number of principles and values rooted in mainstream Western bioethics, such as patient autonomy, informed decision making, truth telling and control over the dying process.^4^ However, emerging research indicates there are significant cross‐cultural differences in attitudes towards end‐of‐life care and decision making,^5^, ^6^, ^7^, ^8^ and the common principles and values that underpin ACP may not be shared or accepted by different cultural groups.

The South Asian ethnic group (e.g. East Indian, Pakistani and Sri Lankan) contains multiple language, religious and other sociocultural identities. This group is the largest visible minority group in Canada^9^ and is one of the fastest growing immigrant communities among other Western countries.^10^ Very little research has been published relating to end‐of‐life care issues for immigrants to Canada in general and South Asian immigrants in particular. Deeper understanding is urgently needed on how ACP is perceived and best approached within the South Asian community.

This study aimed at achieving the following objectives: (i) to gain an understanding of the barriers and facilitators to participating in ACP from the perspective of individuals who identify with the South Asian community; and (ii) to determine the ways in which members of the South Asian community would like to engage in ACP.

## 2 METHODS

### 2.1 Study design

This study was undertaken by members of the South Asian community in Calgary, Alberta, Canada, who graduated from the Patient and Community Engagement Research programme (PaCER) at the University of Calgary.^11^ The PaCER programme trains citizens to become patient engagement researchers to design and conduct health experience research and to work in collaboration with health providers, planners and researchers.

**Figure 1:**
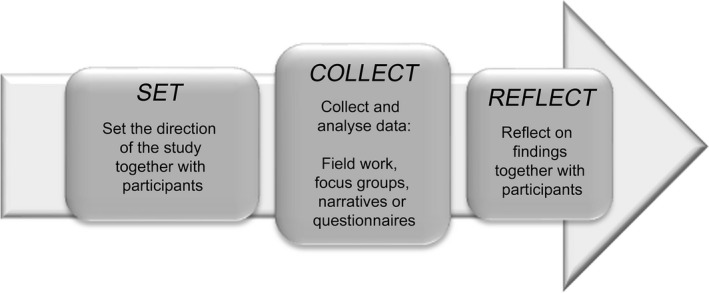
Patient and Community Engagement Research methodological framework^12^.

The PaCER method has a distinct structure defined as SET, COLLECT, REFLECT (Figure 1) to ensure that participants are meaningfully engaged throughout.^12^, ^13^ The method is built on peer‐to‐peer inquiry by researchers with shared experiences. The SET co‐design focus group invites representative patients/community members to become advisors and help set the stage for the study by refining the protocols (e.g. recruitment and locations), questions and data collection. The COLLECT phase generally includes one or more data collection strategies. During the final REFLECT phase, participants are invited to analyse emerging themes together with the researchers and validate study findings.^13^

Four PaCER researchers, who are all women, self‐identify as members of the South Asian community in Calgary and are fluent in Punjabi, Hindi, Urdu and English, designed and conducted the study in collaboration with the University of Calgary research team. They recruited study participants, collected and analysed the data, and translated findings into English.

### 2.2 Participants and recruitment

Individuals who self‐identified with the South Asian community, who were over 18 years of age, who spoke fluent Punjabi, Hindi, Urdu and/or English, and who consented to participate in the research were eligible. We did not explicitly exclude English‐only speakers, but participants needed to be able to converse in the language preferred by the focus group or family.

Participants were recruited to the SET focus group through posters displayed in community places of worship and community centres, through announcements by community leaders at religious ceremonies, and through word of mouth by the PaCER research team in the community. A short interview on the local South Asian radio station was used to recruit participants to the COLLECT family interviews. Participants were recruited to the REFLECT community forum through posters displayed in community centres, and via word of mouth. The REFLECT participants had not participated in earlier phases of the study and had not been exposed to the findings from the SET and COLLECT phases.

### 2.3 Data collection and analysis

#### 2.3.1 SET co‐design focus group

A focus group was held at a local Islamic community centre. To respect cultural norms, the SET focus group included women only (n=15). The discussion took place in the participants' preferred languages (Punjabi and Urdu).

The co‐design focus group began with an explanation of the concept of ACP, followed by the sharing of a personal ACP‐related story by one of the PaCER researchers. A number of guiding questions were then used to explore the concept of ACP with the group (Box 1).

Box 1 **Eliciting experiences with advance care planning**

1 We asked participants how they defined the concept of ACP, but found that many participants did not have a clear understanding of what ACP meant. After the PaCER facilitator tried to explain the concept of ACP, one PaCER started the discussion by sharing her own personal story. She opened the conversation by saying that she did not know anything about ACP until her family was hit by the “cancer bus.” She continued:

“Having recently migrated to Canada, and being in perfect health thus far, ‘death’ was something I simply could not wrap my head around until it actually happened. My son and I were only committed to ‘life’ with our husband and father, and death was never an option.

There is a school of thought that believes that we are the co‐creators of our life, agents who have the power to manifest what happens to us. We did not wish to err by indulging in ‘negative’ thought. We erred instead on hope, joy, and on being together. We did not wish to die daily, no matter what we were told. We chose to die only once.”

We then asked the participants the following SET focus group guiding questions:

- Tell us about a time when a loved one was unable to make his/her own medical decisions.
- Was there anybody to help?
- What helped you get through this?
- What would you have liked to happen?
- Who would initiate or start a conversation about health plans in your family? How and when?

Participants deemed family interviews as most appropriate for the COLLECT phase, based on a shared understanding of the importance of family involvement in health‐care decision making within South Asian culture, and, necessarily, to include male perspectives. Focus group members then helped refine the language to use, recruitment, and the format and guiding questions for the family interviews.

#### 2.3.2 COLLECT phase

Eight family interviews were conducted with a total of 23 participants. Interviews were conducted by pairs of PaCER researchers in the families' residences and in the participants' preferred languages (Punjabi, Hindi, Urdu or English).

The interview format and guiding questions are presented in Table 1. Family interviews started with the sharing of a personal ACP‐related story by a PaCER researcher; this led participants to share their thoughts in the form of stories about their own situations, their families, friends and communities. The telling of stories emerged as a natural tool for sharing ideas on a complex issue (i.e. ACP) within the context of community traditions and practices. Interviews were audio taped and transcribed, and the transcripts were analysed using a unique narrative method^12^ that included bracketing stories, using a story analysis format to identify the context (actors, location and triggers), the plot of the story and the consequences (Appendix 1). Similar story units were then analysed to uncover common degrees of agency (internal and external locus of control), actions (based on plot summaries) and outcomes. The stories were resorted to explore shared meaning through identifying common general “scripts”—recognizable patterns that play out in similar ways throughout many stories recounted by different storytellers, or the same storyteller. Bruner^14^ explained scripts as the “canonical events” which introduce the meaning, or frame of a story. Labov and Waletzky^15^ defined scripts as the referential core of personal narratives.

**Table 1:** COLLECT interview format and guiding questions

COLLECT family interview format
PaCER researcher to share a story around ACP
Guiding questions: What have you done around ACP? Why or why not? What would have helped you in the situation?
Possible prompts: What would you have done if something similar were to happen in your family? What would be most difficult?

ACP, advance care planning.

Supporting quotes from these shared scripts were located and a resulting script summary prepared for discussion in the REFLECT phase. These were then translated into English by the PaCER researchers for the community forum.

#### 2.3.3 REFLECT phase

A community forum open to members of the South Asian community was chosen to allow for sharing of the SET and COLLECT findings with the public, and to invite participants to suggest ways to openly discuss ACP within the community.

The community forum was held at a convenient and accessible local community leisure centre. Participants (n=19) were introduced to the concept of ACP and then split into four small groups. Over the course of the morning, with the help of PaCER facilitators, each group discussed each of the scripts developed from the COLLECT family interviews. Detailed field notes were taken by the PaCER facilitator at each table. By prior consent, members of the ACP CRIO research team and health system were invited to join the forum for a shared traditional lunch. In the afternoon, key findings of the study were presented, and a summary of discussion points from the morning session was reflected back to the large group to test out the ideas generated. This was followed by a group discussion of how ACP could be encouraged within the South Asian community, with field notes taken by PaCER team members throughout the discussion. Community forum data were later analysed by PaCER researchers and PaCER supervisors, drawing on the PaCER researchers' experiences with the previous study phases to interpret and integrate the new data.

### 2.4 Ethical approval

This study was reviewed and approved by the University of Calgary Conjoint Health Research Ethics Board (REB14‐0247).

## 3 RESULTS

Fifty‐seven individuals participated in the study: 15 women in the SET focus group, who identified with three South Asian religious communities: Hindu, Sikh and Muslim; 23 men and women in the COLLECT family interviews (five families who identified with the Muslim community and three with the Sikh community); and 19 men and women in the REFLECT community forum (who identified with the same three religious communities as the SET group) (Table 2). This diverse group of participants ranged in age from 22 to 86 years old, the majority of whom could speak English in addition to Urdu, Punjabi and/or Hindi, and included some individuals who were born in Canada, some recent immigrants (<5 years in Canada) and some individuals who had lived in Canada for 20+ years.

**Table 2:** Study participants

	SET focus group	COLLECT family interviews	REFLECT community forum
Number of participants	15	23	19
Age (years)			
Mean	46	42	N/A^a^
Range	22‐86	22‐72	N/A
Gender, n (%)			
Female	15 (100)	13 (57)	N/A
Male	0 (0)	10 (43)	N/A
Languages spoken, n (%)			
Urdu	11 (73)	15 (65)	N/A
Punjabi	8 (53)	12 (52)	N/A
Hindi	3 (20)	5 (22)	N/A
English	10 (67)	20 (87)	N/A

a As an open community forum, participants' identifying information was not recorded. The age range spanned three generations from young adults in their twenties to older adults in their 70s‐80s with both genders present and all languages spoken and translated as needed by the PaCER researchers.

### 3.1 Learnings from the SET focus group

**Figure 2:**
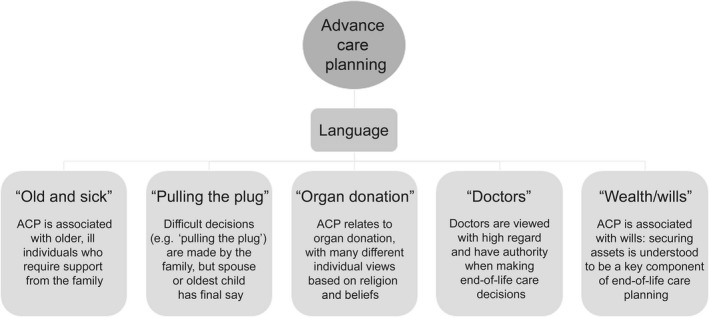
Different understandings of advance care planning

**Figure 3:**
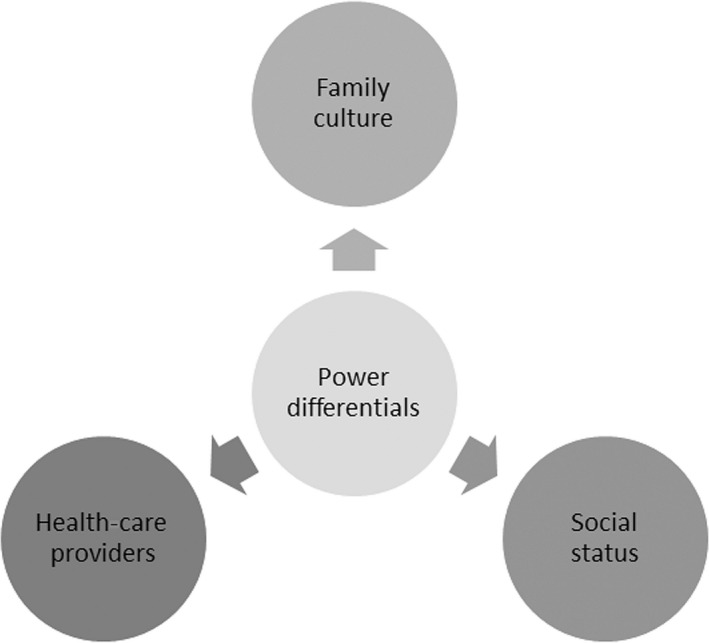
Power differentials within the South Asian community

Three main learnings emerged from the SET focus group:

1. *Importance of language*. There is no literal translation of the phrase “advance care planning” into Hindi, Punjabi or Urdu and, accordingly, focus group participants had a difficult time understanding the meaning of ACP. The concept of planning ahead for future medical decision making did not appear to exist within the community and was foreign to focus group members. There was no shared historical or health service contexts familiar to participants that could be used to draw on examples or describe ACP. Participants had few relevant “mental models” within their familiar practices that could be helpfully used in communicating the concept of ACP. To address this, PaCER researchers spent time finding the “right” words—practicing describing the process of ACP with family and friends prior to the COLLECT phase of the study. Vignettes were used to set the stage and engage participants, and then individuals were invited to share their own personal narratives in a similar matter. Such storytelling proved an important mode of explanation and expression.
2. *Different understandings of ACP*. The meaning of ACP varied from individual to individual based on her personal understanding of the issues. This understanding was shaped by the individual's personal experiences and how they were viewed. Common understandings that emerged are presented in Figure 2. An underlying influence among these themes was family. Families tended to play a key role when making important decisions, such as whether or not to be organ donors, when to “pull the plug,” and where to care for sick elders.
3. *Power differentials*. Many discussions were related to power differentials (Figure 3), which influenced an individual's ability to make end‐of‐life decisions and created a natural dependency on others. Within families, in most cases, the oldest male was responsible for making all important decisions:

Since my husband was the eldest son, he was automatically responsible for taking care of [my mother‐in‐law].

The power differential within families was a naturally understood concept and was a traditional cultural norm. Within the health‐care system, participants described having a high regard for health‐care providers when making end‐of‐life decisions, with doctors given authority over decision making:

In our community, doctors' advice is highly valued and respected … some consider them as God.

Doctors' views are important and we should not ignore them.

There was also a power differential related to social status within the community. Members who were considered “power figures,” such as priests or politicians, were highly respected and became “go‐to” people for discussing important issues or raising awareness of issues:

It would be beneficial if more seminars happened at the temples because people tend to pay more attention to the priests and take them seriously instead of us or you.

### 3.2 Learnings from the COLLECT family interviews combined with the SET focus group

Themes that emerged from the family interviews were plotted onto a story analysis chart (Appendix 1), which facilitated the organization and development of scripts. These scripts were combined with the learnings from the SET focus group and refined into six key scripts:

1. *Foreign ideas*: “When I don't know much about a new foreign idea, I simply cannot make any decisions, and then I ask for more information so I can deal with it.”

Almost all families interviewed were unaware of ACP. It appeared to be a new concept, which they found difficult to grasp (e.g. some families associated ACP only with estate planning such as wills). According to one participant:

It is not a trend in our society to discuss ACP, it is so alien to their minds.

Another participant was under the impression that his will covered his health‐care decisions.

As ACP is a health‐related concept, many participants were surprised that their family physicians (who were considered their primary source when accessing health‐related information) had never discussed it with them:

We have never heard about ACP before, even our doctors have never told us anything about it.

There was a general belief that conversations around ACP should be initiated by health‐care providers:

If healthcare tell us, we will be more comfortable to discuss with them, instead of our family.

- *Cultural norms*: “When anyone in the family is faced with a difficult situation, everyone intuitively knows what their role is and what to do, and then right decisions are just made without us planning ahead.”

Cultural norms significantly influenced participants' perspectives on ACP. Many participants expressed the common belief that planning ahead is not necessary because of close family ties, pre‐defined roles within the family and trust in shared decision making within the family:

We have a good value system within our families and they are not worried that their families will not take care of them.

They are not concerned about their health [because] they have more family ties and believe that family would make the decision for them.

My family will make my health decisions, but we have not talked about it.

I entrust my family to carry out my wishes.

There was a common understanding among families that if a family member became sick, especially an elder, the family/children would naturally take care of them:

I told my doctor that I have kids who will take care of me and I am comfortable with them … I don't need home care.

We see our forefathers take care of their elderly and we do it automatically.

- *Don't talk about that*: “When I anticipate any unfortunate scenario, I am immediately told to shut up and not to dwell upon it, so we don't prepare for problems, we just face them.”

Superstitions (e.g. speaking about death may invoke it) and/or death and dying as taboo discussion topics emerged as barriers to discussing ACP:

We cannot speak of bad things or else we shall make them happen.

We don't plan for death and it is not discussed in our family. We are brought up in such a way that death is not discussed.

We don't like to think that anything will happen to us and, therefore, fail to plan our health decisions.

Because of the common belief that “thinking positively” or “focusing on the positive” was important to delivering positive outcomes, ACP was viewed negatively. Some families were reluctant to discuss this topic and felt they would cross that bridge if and when they came to it:

We don't prepare for problems, we just face them.

However, some generational differences emerged, with some younger participants believing that individuals should be allowed to speak out directly when planning end‐of‐life care decisions.

We do not think of this, although it would be helpful if we did. It would avoid family disputes at painful times.

- *Leave it to God*: “Whenever I am faced with hardships, I turn to God and then I feel peace and contentment.”

Religion figured prominently in thinking about future health‐care planning. Participants strongly believed that God would “take care of everything” if they were to fall ill or something “bad” was to happen:

Faith in God is a strong factor due to which we don't take care of our death and health plans.

God is the One who helps us in every step of our life. He created our body and He helps us in our decision for life. He has strong impact on our decision.

Attitudes towards health‐care planning tended to be fatalistic, believing that “one has to surrender to the higher wills of God”:

We don't plan about death and severe health conditions as we believe it is not in our control. Discussing and preparing for it puts us in control rather than God and the higher power.

God is the one who makes the final decision as to when it's time to go.

Not everyone agreed with this view, however, with a couple of dissenting opinions voiced:

Our people don't know about their religion and are narrow minded. They don't have all the information before making a decision.

I believe in science and technology, and don't let religion come in the way when making decisions.

- *Too busy with life*: “I am so busy with making a living in Canada that I have no time to think of anything else that might be important in my life.”

Dealing with the many challenges associated with emigrating from South Asia to Canada emerged as a barrier to becoming informed about and participating in ACP. Finding affordable housing, securing employment, sometimes learning a new language and enrolling children in schools (among other pressures), all while adapting to a new culture, left little time for ACP:

There are so many challenges here, that sometimes I forget my name.

I wake up at 5 am and come home tired, have to cook, eat, etc.

How will we have this thought in our mind when we are having this life stress?

We are too busy to think about ACP.

Never took it very seriously as our priorities are education and economics.

Most participants described focusing their energies on “tangible” activities (e.g. finding a home and getting a job). In contrast, ACP was perceived as a vague and irrelevant activity that would burden them with additional worries about things that may or may not happen.

- *Wealth vs health*: “When I fall ill, I make sure that my finances are in order, so that my family doesn't fight over it.”

Planning for the end of life was more often associated with wealth distribution than with health‐care planning. Participants were much more likely to have prepared a will and/or thought about the allocation of their assets than to have thought about their preferences for future health care:

What would happen if something happens to me, do we have to write a will? What will happen to my bank account?

Many people don't include their health decisions in their will, it's all about who gets what.

Wealth decisions are more important than health decisions.

Many families felt that having their finances in order helped them feel better prepared to face “the worst.” They also saw value in having a will for the purposes of conflict resolution:

It is better to have a will to avoid conflict between families.

### 3.3 Learnings from the REFLECT community forum

Three key themes emerged from the REFLECT phase of the study:

1. *Got it!!!*

We were amazed to witness that, after we introduced ACP with the scripts, stories and quotes we had developed and our purpose for gathering, participants in the REFLECT phase immediately understood and shared their excitement by saying, “Such a great thing we are doing by bringing this awareness and that more should be done.”

- *Consolidation of experiences around end of life*

Forum participants learned from each other by listening to each other's experiences, particularly of encounters with illness and death of loved ones. Regardless of belonging to different cultures, religions and language groups, participants engaged in a sharing of knowledge, feelings, grievances and ideas. This sharing was a strong facilitator of the group's learning, given their cultural and religious differences.

- *Community capacity*

Community forum participants had a high level of participation, were actively engaged in their health and were open and receptive to exploring new ideas. There appeared to be a strong community capacity for creating awareness on health issues and implementing new ideas—despite the variations in culture the community demonstrated a strong connection to traditional values and community norms: there are strong family traditions, there are close communal ties, and people feel a high degree of responsibility for each other. Building on family and community capacity will be crucial to creating more dialogue around ACP, and we posit that change will likely be more effective and sustainable if it is initiated within the community and implemented by community members themselves, based on their existing cultural practices and norms.

- *Community recommendations*

We found that participants were eager to learn more about ACP and recommended several strategies for engaging their community (Table 3). A number of suggestions were aimed at promoting document completion, for example make forms mandatory, put forms online that can be submitted electronically to the provincial health‐care system, complete forms on iPads in physician waiting rooms and synchronize ACP document completion with drivers' license renewals. Some participants suggested the health‐care system should take more initiative in educating and informing the community around ACP, and that a formal letter from the provincial health‐care system or government would be taken seriously. Participants were also keen to see their ACP information be accessible to health‐care providers during times of need, for example via an electronic medical record and linked to their personal health number. Finally, many participants emphasized that the provincial health‐care system and health‐care providers should be aware of common South Asian religious beliefs.

**Table 3:** Participant recommendations for engaging South Asian community members in ACP

Recognize and build on community capacity	Find ways to capitalize on the community capacities (family networks, community ties and shared norms) that already exist.
Inform the community through forums and seminars	Hold forums or seminars in participants' native languages at religious institutions and community centres. Temples or mosques are often consulted to obtain information about community events.
Involve religious leaders in ACP discussions	Religious leaders are considered at the top of the social hierarchy, are highly respected and trusted and are in a position to raise awareness of important issues in the community.
Include family members in ACP discussions	Important decisions are shared within the family. Conversations around ACP need to be multigenerational and include the entire family.
Respect cultural norms	View cultural norms as positive potential to build upon, rather than barriers. Cultural and community norms should be considered an asset and a powerful facilitator of natural decision‐making processes.
Encourage doctors to initiate the discussion	ACP conversations should be initiated by health‐care providers at the primary‐care level, as most community members have family physicians. Many participants would be more comfortable talking with their doctors about end‐of‐life care planning than with their friends and family.
Translate information materials	Forms/resources should be translated into various languages, and be distributed throughout the community in doctor's offices, at registries, in community centres, or in religious institutions and on the Internet.

ACP, advance care planning.

## 4 DISCUSSION

This study explored South Asian community members' perspectives towards ACP using a patient engagement research model and peer‐to‐peer inquiry. To our knowledge, this is the first study to explore South Asians' perspectives, in Canada, towards ACP. A limited number of studies have explored attitudes towards end‐of‐life care,^16^, ^17^, ^18^ which occasionally encompassed aspects of ACP, and a few studies have explored other sociocultural perspectives on ACP.^5^, ^19^, ^20^

Several themes emerged describing barriers to participating in ACP, for example the foreign concept of planning for death/dying, the taboo nature of discussing death and dying, delegation of decision making to family members, fatalistic attitudes towards health‐care planning and immigration challenges. These themes for the most part are consistent with the literature. A lack of open discussion about death and dying (partly related to superstitions around discussing death) appears common,^19^, ^21^, ^22^ as are religious beliefs pertaining to lack of control over death.^19^, ^22^ Language barriers were also reported in two studies.^21^, ^22^ Universal to almost all studies was the role of family members in decision making,^16^, ^17^, ^18^, ^19^, ^22^ and how power differentials vary with culture is also well described in the literature.^23^

While a lack of knowledge surrounding ACP and low advance directive completion rates have been reported in this population,^16^, ^17^, ^19^, ^24^ there is increasing evidence, including from this study, that South Asian individuals are interested in participating in ACP. Sharma et al. and Rao et al. reported positive attitudes among study participants towards advance directives, with many expressing a belief that they could reduce decision‐making burden on family members.^16^, ^17^ A survey of Asian Indian Hindus living in the USA indicated that 44% of respondents desired to complete an advance directive.^24^ In our study, participants also described a willingness to complete documentation and suggested ways to introduce this to the community.

### 4.1 Strengths and limitations

The strength of this study was its user‐led focus, with researchers from the South Asian community conducting the research and the entire time being advised by others from that community. These researchers were in a position to establish trusting relationships with potential participants and thus were able to engage an otherwise difficult‐to‐reach population. Additionally, focus groups and interviews were conducted in participants' native or preferred languages, reducing the potential for misinterpretation. With the level of understanding made possible through this peer‐to‐peer inquiry, we were able to use narrative to build a conceptual bridge between ACP, which has emerged from Western health‐care systems, and the South Asian cultures. Such sharing of narratives to bridge cultural differences has been described,^25^, ^26^, ^27^ but in our experience is still under‐utilized in clinical practice.

The research reported here has several limitations. First, the South Asian community is extremely diverse and includes individuals emigrating from a number of different countries with different degrees of assimilation/acculturation, speaking many different languages, and being of different faiths and cultures. This study describes a sample of the attitudes held by members of the South Asian community in Calgary, and we cannot seek to generalize these results to the overall South Asian community there, elsewhere or to other sociocultural groups in Calgary. Furthermore, while we did not explicitly exclude English‐only speakers, we did not encounter any participants or families who only spoke English, so their perspectives are missing from this study. Second, the SET focus group was comprised of women only. We lacked male PaCER researchers who could have conducted male‐only focus groups. However the COLLECT and REFLECT phases did allow us access to mixed‐gender family groups. Finally, as we collected limited demographic information in the SET and COLLECT phases, we do not have complete details for all participants, such as education level or years of living in Canada. We are aware that time in Canada ranged roughly from two to 38 years, with a few participants who were born in Canada, but without more comprehensive demographic data we are limited in our ability to better characterize the study population.

## 5 CONCLUSIONS

Many health‐care providers are aware of the need for “cultural sensitivity” in medical interactions.^28^, ^29^, ^30^ Our study suggests that when seeking to engage South Asian community members in ACP, health‐care providers may need to inquire after and recognize just how “foreign” a concept ACP can be and to try using stories from their own experiences and other patients' experiences to help foster understanding.

## CONFLICTS OF INTEREST

None to declare.

## APPENDIX 1

### Story Analysis Chart

#### Story analysis

**Table:**

Title
Story Context
Plot *(Triggers; strategies)*
Outcomes/Consequences/Lessons *(Researchers' summary)*
Storyteller's reaction to telling the story/what they learned: ___________________ Your reaction to the story, what you learned:_______________________________
